# Special Issue “Recent Advances in Molecular Mechanisms of Human Papillomavirus Family (HPV) Induced Oncogenesis”

**DOI:** 10.3390/ijms27135865

**Published:** 2026-06-29

**Authors:** Subhasis B. Biswas

**Affiliations:** Department of Medical and Molecular Sciences, College of Health Sciences, University of Delaware, Newark, DE 19716, USA; biswassb@udel.edu

## 1. Introduction

Human papillomavirus (HPV) represents one of the most clinically significant oncogenic viruses affecting human populations worldwide. Although HPV has historically been synonymous with cervical cancer, decades of molecular and epidemiological research have revealed that this small double-stranded DNA virus is causally linked to a much broader spectrum of malignancies, including anogenital, penile, anal, head and neck, and sinonasal cancers. The more than 200 identified HPV genotypes encompass a wide range of oncogenic potential, with high-risk types such as HPV16 and HPV18 accounting for the majority of HPV-associated cancers globally. Despite the remarkable success of prophylactic HPV vaccines, HPV-driven malignancies remain a substantial contributor to cancer morbidity and mortality, particularly in resource-limited settings and among populations with inadequate vaccination coverage.

The molecular mechanisms that underlie HPV-induced oncogenesis have been the focus of intense investigation. Central to this process are the viral oncoproteins E6 and E7, which inactivate the tumor suppressors p53 and retinoblastoma (pRb), respectively, thereby driving unchecked cellular proliferation and genomic instability. Equally important are the early viral proteins E1 and E2, which orchestrate viral DNA replication and regulate oncogene expression, and the less well-studied E5 oncoprotein, which modulates growth factor signaling. More recent research has broadened our view to encompass epigenetic reprogramming, microRNA dysregulation, HPV genomic integration into host DNA, and remodeling of the tumor immune microenvironment. Together, these mechanisms contribute to the progression from viral infection to malignancy.

This Special Issue of the *International Journal of Molecular Sciences*, entitled “Recent Advances in Molecular Mechanisms of Human Papillomavirus Family (HPV) Induced Oncogenesis,” brings together thirteen peer-reviewed contributions, seven original research articles, and six reviews to provide a contemporary and multidisciplinary view of HPV biology and HPV-related cancer. The papers cover molecular virology, clinical diagnostics, biomarker development, therapeutic approaches, and newer concepts. This editorial summarizes the main findings of each contribution, places them in the context of recent developments elsewhere in the field, and highlights how, taken together, they mark meaningful progress in our understanding of HPV-induced oncogenesis.

## 2. Molecular Virology of HPV DNA Replication and Transcription

Three contributions from our own laboratory addressed fundamental aspects of how HPV proteins interact with viral DNA to orchestrate replication, transcription, and oncogenesis.

Evande, Rana, Biswas-Fiss, and Biswas (contribution 1) provided a comprehensive review of protein–DNA interactions that regulate HPV DNA replication, transcription, and oncogenesis. The review emphasized the central roles of the viral E1 helicase and E2 transcription/replication factor in the assembly of the pre-initiation complex at the origin of DNA replication. **It also summarized** how E2 governs the transcription of the E6 and E7 oncogenes by occluding transcription factor access to the p97/p105 promoters. A particularly useful contribution of this review was its articulation of a sequence-based classification scheme in which single-nucleotide variations within the consensus E2 binding sites (particularly BS3) distinguish high-risk from low-risk HPV genotypes. **This framework allows** the oncogenic potential of uncharacterized HPV types to be predicted from genome sequence alone. By drawing explicit mechanistic parallels between HPV origin activation and the well-studied E. coli DnaA/DnaB replication initiation system, and by integrating biochemical, biophysical, and structural data, the article framed E1–E2–DNA interactions as a promising target for future antiviral and anticancer strategies.

Yilmaz, Biswas-Fiss, and Biswas (contribution 2) reported a detailed quantitative analysis of how the full-length HPV11 E2 protein recognizes and binds to the four E2-binding sites within the viral origin of replication. Using purified full-length E2 protein rather than isolated DNA-binding domains or GST fusions used in earlier work, these studies established a clear affinity hierarchy (BS4 > BS1 > BS2 > BS3; Kd values ranging from 4.8 to 10.2 nM). **The lower affinity for BS3** plausibly makes binding at this site the rate-limiting step in origin activation. The work also dissected the contributions of the four-nucleotide spacer and the immediately flanking sequences to target discrimination: binding site variants with altered spacer lengths of three, five, or six nucleotides failed to form detectable E2–DNA complexes, whereas substitutions in the flanking hexamers modulated affinity more subtly. Thermodynamic analysis across a range of temperatures and ionic strengths indicated that E2–DNA recognition is driven predominantly by hydrophobic rather than ionic interactions. Atomic force microscopy of E2 bound to a 1088-bp HPV11 origin fragment revealed large multimeric protein–DNA assemblies localized at the BS1/BS2/BS3 cluster, providing a structural rationale for the coordinated regulation of replication and transcription by multiple cooperating E2 dimers.

**Figure 1:**
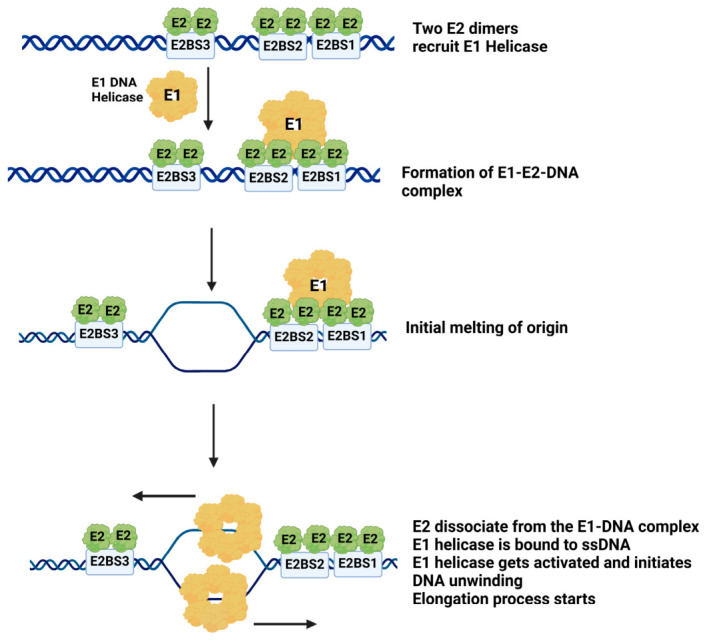
**Proposed model for E1–E2 complex assembly and initiation of HPV DNA replication.** Two E2 dimers first bind to cognate E2 binding sites (E2BS1 and E2BS2) within the origin and recruit the E1 helicase to form the E1–E2–DNA complex. Binding of E1 together with E2 at the cluster of E2 binding sites promotes localized melting of the AT-rich region, after which E2 dissociates and double-hexameric E1 bound to ssDNA initiates bidirectional DNA unwinding. Reproduced from Rana et al. (contribution 3) under the Creative Commons Attribution (CC BY 4.0) license.

Building on these findings, Rana, Yilmaz, Biswas-Fiss, and Biswas (contribution 3) investigated how E2 recruits the E1 DNA helicase to the origin of replication. In parallel, they presented a comprehensive biochemical characterization of the purified full-length HPV16 E1 protein. The protein showed intrinsic ATPase activity (Vmax ≈ 37 nmol/min/mg) that was stimulated approximately two-fold by long circular M13mp19 ssDNA but not by short oligo-dT30 or dsDNA substrates, consistent with ATP-coupled translocation along single-stranded DNA. Using a fluorescently labeled partial duplex substrate, they further confirmed concentration-dependent strand-displacement helicase activity, reaching roughly 28% unwinding at the highest enzyme concentrations tested. Electrophoretic mobility shift assays with purified full-length E1 and E2 then produced an important mechanistic result: E1 alone did not bind origin DNA with detectable affinity, even at high protein concentrations. Instead, E2 was required to recruit E1 and form a stable E1–E2–DNA ternary complex, and at least two E2 binding sites were needed for this higher-order assembly. Critically, complex formation occurred equally well with DNA probes that lacked the putative E1 binding site, directly challenging the prevailing BPV-derived model in which a dedicated E1 binding sequence is required for helicase loading. The authors propose an alternative model, reminiscent of DnaC-independent DnaB loading in E. coli, in which E2 dimers act as the primary organizers of the pre-initiation complex and the AT-rich region near the E2 cluster serves as the site of initial origin melting (Figure 1). These findings refine our understanding of the earliest steps of HPV replication and highlight the E1–E2 interaction as a mechanistically rational antiviral target.

Together, these three contributions establish a cohesive molecular framework for HPV DNA replication and transcription, and underscore how detailed biophysical characterization of viral protein–DNA complexes can inform the rational design of small-molecule inhibitors that disrupt viral replication at its earliest and most vulnerable step.

## 3. Viral Oncoproteins, Signaling, and Epithelial–Mesenchymal Transition

Raffa et al. (contribution 4) examined the role of the HPV16 E5 oncoprotein in preneoplastic anal epithelial lesions. Anal squamous cell carcinoma (SCCA) is a rare but clinically important HPV16-associated malignancy whose pathogenesis remains incompletely understood. Expanding on earlier work in keratinocyte and cervical lesion models, the authors quantified transcripts for 16E5, FGFR2c, and EMT-related transcription factors (Snail1, Snail2, ZEB1) in HPV16-positive anal cytology samples stratified by cytopathologic grade. A significant positive correlation between HPV16 E5 expression and mesenchymal FGFR2c expression was observed across all HPV16-positive samples. This relationship, along with the strong associations between HPV16 E5/FGFR2c and the EMT transcription factors Snail1 and ZEB1, was most evident in the non-high-grade (Group A) samples. In contrast, high-grade (HSIL, Group B) samples displayed uniformly elevated EMT-factor expression, suggesting that EMT activation at this stage may be largely uncoupled from HPV16 E5 and FGFR2c expression. The authors interpret this stage-dependent pattern as evidence that 16E5 acts chiefly during the early transition from normal epithelium to low-grade dysplasia by inducing the FGFR2 isoform switch and priming the EMT program, whereas in HSIL the EMT machinery has already been established and becomes 16E5-independent. A bioinformatic interactome analysis using the BioGRID database further identified the Ca^2+^ channel TRPA1 among the 16E5 protein interaction partners, suggesting a possible post-translational functional interplay between 16E5 and TRPA1 in modulating FGFR2c signaling. These findings support a model in which 16E5 contributes specifically to the earliest steps of anal carcinogenesis and may represent a candidate target for preventive as opposed to late-stage therapeutic strategies against HPV16-positive SCCA.

## 4. HPV Genomic Integration, DNA Methylation, and microRNA Regulation

Li and Li (contribution 5) contributed a comprehensive review of HPV integration as a hallmark event in cervical carcinogenesis. The authors walked the reader through the multi-step oncogenic cascade:viral entry via L1/L2 proteins, immune evasion, persistent infection, activation of DNA damage response pathways, integration at vulnerable host genomic sites (e.g., 3q28 and 8q24) through microhomology-mediated end joining, and subsequent genomic rearrangement, epigenetic remodeling, and immune microenvironment reprogramming. This synthesis positions HPV integration not only as a molecular biomarker of increasing clinical utility but also as a conceptual framework for understanding how a viral infection becomes an irreversible oncogenic event.

Pulliero et al. (contribution 6) provided a systematic review of microRNA (miRNA) expression and DNA methylation changes in HPV-related cervical cancer. Screening the literature from 2018 through 2023, they identified miR-124 and FAM19A4/miR-124-2 methylation among the most consistently altered epigenetic markers in cervical carcinogenesis. The authors argued that FAM19A4/miR-124-2 methylation, in particular, shows strong promise for clinical triage distinguishing lesions requiring immediate surgical intervention from those suitable for conservative “wait-and-see” management. In the era of precision medicine, such biomarkers could help reduce overtreatment of low-grade disease while ensuring timely intervention for high-grade lesions.

Complementing these reviews, Pruski et al. (contribution 7) conducted a clinical evaluation of methylation testing as a new tool for diagnosing and risk-stratifying squamous intraepithelial lesions. In a cohort of 108 women undergoing cytology, HPV genotyping, colposcopy, biopsy, and methylation testing, a negative methylation test was significantly associated with a final diagnosis of low-grade rather than high-grade disease. Importantly, the authors estimated that in approximately 85.7% of patients initially classified as high-grade, major cervical surgery could have been avoided had a negative methylation test been used to guide management. This study supports integrating methylation-based assays into cervical cancer screening algorithms.

## 5. Clinical Predictors, Genotyping, and Proteomic Biomarkers

Medina Bueno et al. (contribution 8) reported an observational study of 189 women with abnormal cervical cytology in Peru, examining predictive factors for cervical intraepithelial neoplasia grade 2–3 (CIN2–3) across HPV genotypes. Multiple logistic regression identified age ≥ 30 years (adjusted OR 4.50) and HPV16 infection (adjusted OR 4.19) as the strongest independent predictors of high-grade disease. HPV18 was relatively rare in this cohort and was not significantly associated with CIN2–3, whereas high-risk types other than HPV16/18 showed an inverse association. The study reinforces the dominant oncogenic role of HPV16 and supports integrating genotyping, cytology, and age into risk-stratified screening algorithms, particularly in resource-limited settings.

Bober et al. (contribution 9) applied a label-free quantitative proteomics workflow combining nano-HPLC ESI-MS ion-trap analysis with MALDI-TOF/MS screening to urine samples from women with CIN3 and healthy controls. Of 476 proteins identified and quantified, 48 were differentially expressed between groups. Gene set enrichment analysis against the KEGG database implicated the extracellular matrix (ECM) receptor interaction pathway as the most strongly dysregulated (normalized enrichment score −1.64, *p* = 0.026), with 13 ECM-related proteins (HSPG2, COL6A1, COL6A3, SPP1, THBS1, TNC, DAG1, FN1, COMP, GP6, VTN, SDC1, and CD44) consistently down-regulated in the CIN3 group. Among these, only HSPG2 (perlecan) reached individual statistical significance, with the remaining proteins contributing collectively through pathway-level enrichment. On the MALDI-TOF/MS side, a genetic algorithm classifier trained on ten discriminating mass peaks correctly recognized 95% of samples (with 51–52% cross-validation performance), supporting the feasibility of rapid mass-spectrometric screening of urine. Taken together, the results suggest that ECM remodeling possibly initiated by disruption of HPV-relevant proteoglycans such as HSPG2, which is also implicated in initial viral attachment, contributes to the pathogenesis and progression of high-grade cervical lesions. They also highlight urinary proteomics as a promising noninvasive approach for cervical cancer biomarker discovery.

## 6. Therapeutic Strategies and Vaccine Development

Cakir et al. (contribution 10) explored the therapeutic potential of *Ficus carica* (fig) latex against HPV-positive cervical cancer cell lines. Notably, fig latex showed a selective cytotoxicity profile, inhibiting the growth of HeLa (HPV18+), CaSki (HPV16+), and C33A (HPV-negative) cervical cancer cells with comparable IC_50_ values of approximately 106–110 µg/mL. Normal human cervical keratinocytes (HCKT1) remained essentially unaffected across the same concentration range, providing a therapeutic window that strengthens the rationale for further development. RNA-Seq transcriptome analysis of HPV16- and HPV18-positive cells treated with fig latex revealed consistent upregulation of genes involved in Class I MHC–mediated antigen presentation and antigen processing via ubiquitination and proteasomal degradation, including RPS27A, RNF111, CUL5, FBXO4, FBXL4, TRIP12, and CALR. Western blot analysis confirmed increased MHC class I protein expression in HeLa cells following treatment. Because HPV16 E5 is known to suppress surface MHC class I transport as an immune-evasion strategy, these findings raise the interesting possibility that fig latex, or specific components derived from it, could partially reverse E5-mediated immune evasion and enhance T-cell recognition of HPV-infected cells. This mechanism of action could complement existing therapeutic and immunotherapeutic strategies, particularly for early-stage HPV disease.

Wei et al. (contribution 11) reviewed the multidimensional role of HPV in penile cancer (PC), a rare but aggressive male malignancy characterized by early lymph node metastasis and poor prognosis. The review comprehensively summarized virus–host genome integration patterns, genetic alterations, epigenetic regulation (methylation and microRNA modification), and tumor immune microenvironment remodeling in HPV-associated PC. The authors also critically evaluated current and emerging HPV vaccination strategies for the prevention and treatment of penile cancer, arguing that expanded gender-neutral HPV vaccination programs have significant public health potential beyond cervical disease.

## 7. Emerging Frontiers: HPV-Independent Cancers and Sinonasal Disease

Hurjui et al. (contribution 12) provided a timely review of HPV-independent cervical cancer, an entity that accounts for approximately 5–11% of cervical carcinomas and presents distinct histopathological, molecular, and clinical features. Unlike HPV-associated tumors, which depend on E6/E7-mediated inactivation of p53 and pRb, HPV-independent cervical cancers, including gastric-type, clear-cell, mesonephric, and endometrioid adenocarcinomas, are driven by mutations in genes such as TP53, PIK3CA, KRAS, STK11, and PTEN. These tumors are frequently misclassified as endometrial in origin, tend to present at more advanced stages, and generally respond less favorably to conventional chemotherapy and immunotherapy, in part because of an immune-cold tumor microenvironment. The authors highlight PI3K/mTOR and KRAS inhibition as rational therapeutic avenues and call for improved diagnostic algorithms to address this clinically challenging subset of cervical cancer.

Guzmán-Romero et al. (contribution 13) turned attention to sinonasal inverted papilloma (SNIP), a benign but locally aggressive neoplasm of the nasal cavity and paranasal sinuses with a notable tendency toward malignant transformation and recurrence. Their review integrated histological features, clinical manifestations, and treatment approaches with emerging insights into the contributions of chronic inflammation, environmental factors, and HPV infection to SNIP pathogenesis. Particular attention was given to the tumor microenvironment, including CD4+ and CD8+ lymphocyte infiltration, macrophage polarization, and increased expression of matrix metalloproteinases MMP-2 and MMP-9, as well as to HPV-associated epigenetic alterations. The work broadens the scope of HPV-associated oncogenesis beyond the anogenital and oropharyngeal tracts.

## 8. Synthesis and Outlook

**Figure 2:**
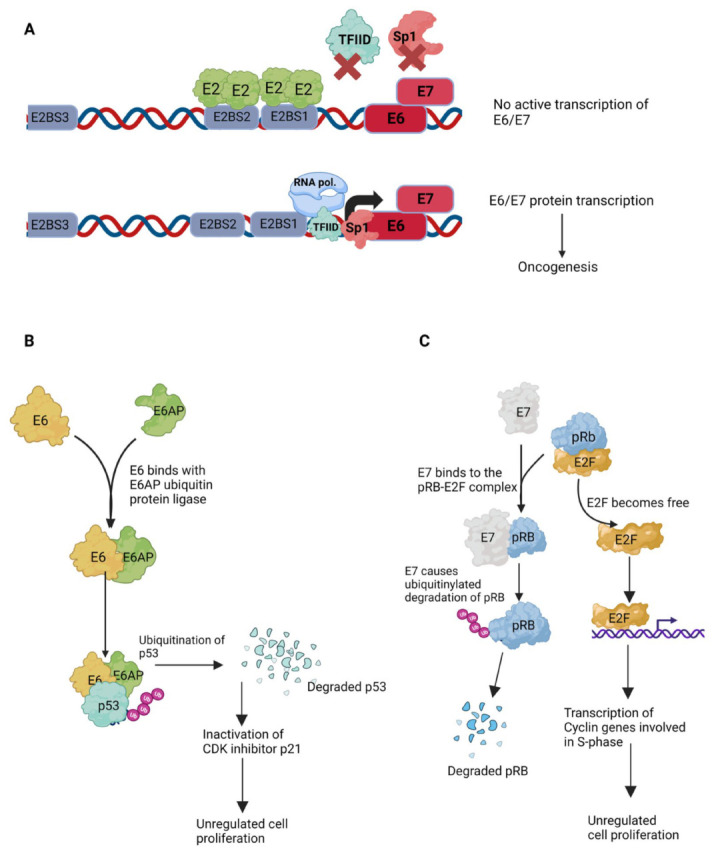
**The pivotal regulatory role of the E2 protein and the principal mechanisms of HPV-driven oncogenesis.** (**A**) E2 binding to BS1 and BS2 **flanks** the E6/E7 promoter and blocks access of host transcription factors (Sp1, TFIID), silencing oncoprotein expression; loss of E2 (typically upon viral integration) permits promoter activation and E6/E7 transcription. (**B**) The E6/E6AP ubiquitin ligase complex ubiquitinates and degrades p53, inactivating the p21-mediated G1 checkpoint. (**C**) E7 targets pRb for **ubiquitin-dependent** degradation, releasing E2F transcription factors and driving unregulated entry into S-phase. Reproduced from Evande et al. (contribution 1).

The thirteen contributions assembled in this Special Issue collectively illustrate how the study of HPV-induced oncogenesis has matured into a truly multidisciplinary field. At the most fundamental level, detailed biochemical and biophysical dissection of HPV E1–E2–DNA interactions (contributions 1–3) continues to refine our mechanistic understanding of viral replication and offers new opportunities for antiviral drug discovery. At the interface of virology and cell biology, studies of E5-driven signaling and EMT (contribution 4) highlight the importance of “secondary” oncoproteins that act alongside E6 and E7 to shape the early tumor phenotype. The central logic that ties replication-level regulation to downstream oncogenic transformation is captured in Figure 2: E2 normally blocks the E6/E7 promoter and represses oncoprotein expression. Upon loss or dysregulation of E2, most often through integration of the viral genome into the host chromosome, E6 and E7 are unleashed and drive the ubiquitin-dependent degradation of p53 and pRb, unlocking unchecked proliferation and genomic instability.

Overall, studies show that HPV infection progresses to malignancy through potentially reversible molecular changes, including genomic integration, epigenetic changes via miRNAs and DNA methylation, and proteomic alterations. These changes can be targeted both diagnostically and therapeutically. Clinical and epidemiological research also shows that integrated testing, combining cytology, genotyping, methylation, and age-based risk analysis improves the precision of cervical cancer screening and reduces overtreatment.

Therapeutically, this Special Issue identifies two main findings: first, novel agents such as *Ficus carica* latex demonstrate immunomodulatory potential; second, HPV vaccines are increasingly effective not only for cervical cancer prevention but also for treating other diseases, such as penile cancer. Additionally, reviews of HPV-independent cervical cancer and HPV-associated sinonasal inverted papilloma underscore the importance of understanding both HPV-driven and HPV-independent pathways of tumor development.

The contributions collected here also arrive at a moment of unusually rapid external progress in the field. On the therapeutic front, Eerkens et al. [1] recently reported a phase II trial of Vvax001, a replication-incompetent Semliki Forest virus vector encoding HPV16 E6 and E7. In this trial, 50% of patients with HPV16-positive CIN3 achieved histopathologic complete response, and 63% cleared HPV16 after three immunizations. These results suggest that many women with high-grade lesions may eventually avoid loop excision surgery. These clinical findings align with the biomarker-driven triage strategies advocated by Pulliero et al. (contribution 6) and Pruski et al. (contribution 7) in this Special Issue. Chen et al. [2] have since extended the therapeutic vaccine paradigm to metastatic disease by reporting a stimuli-responsive STING-activating nanovaccine **carrying HPV16 E7 protein**. This vaccine achieved 71% survival in a mouse model of metastatic HPV-associated lung cancer as monotherapy, and 100% survival in combination with checkpoint blockade. These results reinforce the importance of the immunity-restoring mechanisms uncovered by Cakir et al. (contribution 10) and the vaccine strategies reviewed by Wei et al. (contribution 11). In parallel, long-term follow-up of adoptive cell therapy trials has demonstrated decade-long complete remissions in metastatic cervical cancer following a single tumor-infiltrating lymphocyte infusion. Ongoing trials of HPV-specific T-cell-receptor-engineered T cells show activity across cervical, anal, head-and-neck, and esophageal HPV-associated cancers [3]. Epidemiologically, the most important development is Australia’s position as the first country on a clear trajectory toward WHO-defined cervical cancer elimination (incidence <4 per 100,000 women annually). Modeling projects suggest that this threshold may be crossed as early as 2028. Notably, zero new cervical cancer cases in women under 25 were reported for 2021, a direct result of nearly two decades of high-coverage HPV vaccination and the transition to primary HPV-based screening [4]. Taken together, these external developments provide the molecular advances documented in this Special Issue with a clearer clinical and public health trajectory.

In summary, several priorities emerge from these collective findings. First, there is a need for a more detailed study of HPV replication and transcription machinery to enable the development of targeted antivirals. Second, biomarkers based on integration, methylation, and miRNA require validation in large and diverse cohorts before clinical use. Third, a focus on tumor-specific molecular profiling is needed to identify HPV-independent cancer pathways and improve the precision of therapies. Fourth, ensuring global access to HPV vaccination and screening remains essential to realizing the preventive promise of molecular research.

It has been a privilege to serve as Guest Editor for this Special Issue. I am grateful to the authors, reviewers, and the Editorial Office of the *International Journal of Molecular Sciences* for their support. I hope this collection becomes a valuable resource for clinicians, students, and researchers and inspires further study of the molecular mechanisms underlying HPV-related and HPV-independent cancers.
